# A Systematic Review of the Literature and Perspectives on the Role of Biomarkers in the Management of Malnutrition After Allogeneic Hematopoietic Stem Cell Transplantation

**DOI:** 10.3389/fimmu.2020.535890

**Published:** 2021-01-06

**Authors:** Enrico Morello, Milena Giovanna Guarinoni, Francesco Arena, Marco Andreoli, Simona Bernardi, Michele Malagola, Alessandro Turra, Nicola Polverelli, Domenico Russo

**Affiliations:** ^1^ Unit of Blood Disease and Stem Cell Transplantation, Chair of Hematology, University of Brescia, ASST Spedali Civili, Brescia, Italy; ^2^ University of Brescia, Brescia, Italy; ^3^ Nutritional Service, ASST Spedali Civili, Brescia, Italy

**Keywords:** malnutrition (MeSH), biomarker, graft *versus* host disease, citrulline, insulin growth factor-1 (IGF-1)

## Abstract

Malnutrition is a common problem after allogeneic hematopoietic stem cell transplantation (allo-HSCT) and could impair immune function. Immune dysfunction after allo-HSCT may be linked with infections, GVHD, and relapse and negatively affect the outcome. Aim of this review was to identify malnutrition biomarkers, potentially useful for immune-system monitoring, in the setting of allo-HSCT. After a systematic search, no satisfying biomarker was found, except for citrulline. Citrulline could be useful in monitoring gastrointestinal function after allo-HSCT and its role in the complex relationship with immune-system function ought to be better explored. A multi-omics approach, including biomarkers and PRO (patient reported outcomes) is, in our opinion, the optimal way to study the relationship between malnutrition and transplant outcomes.

## Introduction

Malnutrition is common in patients suffering from cancer since 20 to 70% of them experience undernutrition and about 10 to 20% of the deaths are related to malnutrition (1).

In hematologic patients, malnutrition frequently develops during treatment, particularly in the case of patients receiving chemo-radiotherapy (2) regimens for allogeneic hematopoietic stem cell transplantation (allo-HSCT) (3).

As is known, in the allo-HSCT a chemo-radiotherapy conditioning regimen is followed by healthy donor hematopoietic stem cells (HSCs) infusion and immune-suppression to control graft rejection and graft *versus* host disease (GVHD).

Before allo-HSCT, most patients present a good nutritional status, defined according to SGA (Subjective Global Assessment) and only 23% of them are malnourished (4). Nevertheless, according to the data published by De Defranchi and Colleagues, 60% of patients discharged after transplant show different degrees of malnutrition (5, 6).

The frequent adverse events associated with the conditioning chemo-radiotherapy (oral mucositis, nausea, vomiting, diarrhea, dysgeusia, and psychological depression), together with prolonged hospitalization are the main reasons for this severe and rapid nutritional decline. Therefore, the management of their nutritional status is crucial for a better patient’s care, quality of life, and hospitalization and for transplant procedure cost itself. Considering the indirect effects that the nutritional status may have on transplant-related outcomes, improving the patient’s nutritional status may reduce the incidence of infections and acute or chronic graft *versus* host disease (GVHD), may improve hematological and immunological recovery and, thus, may increase the long-term overall survival by reducing the non-relapse mortality (NRM) (1, 7–9).

The term malnutrition includes different metabolic conditions ranging from a reduced introduction of nutrients and simple weight loss to sarcopenia or cachexia (10). These clinical entities are often present at the same time, while the etiology and pathogenesis may be different.

Cachexia is a severe complex syndrome, tightly associated with an underlying chronic inflammation, often present in patients with cancer. Fearon and Colleagues defined cachexia as: “a multifactorial syndrome characterized by an ongoing loss of skeletal muscle mass (with or without loss of fat mass) that cannot be fully reversed by conventional nutritional support and leads to progressive functional impairment” (11). Cancer cachexia is clinically characterized by the continuous sequence of three stages: pre-cachexia, cachexia, and refractory cachexia. Pre-cachexia is defined by a small loss of body weight (<5% loss of stable body weight), with specific clinical (e.g. anorexia) and metabolic signs and symptoms (e.g. impaired glucose tolerance). Therefore, cachexia can be clinically diagnosed when “patients have more than 5% loss of stable body weight over the previous 6 months, or a body-mass index (BMI) less than 20 kg/sqm and ongoing weight loss of more than 2%, or sarcopenia and ongoing weight loss of more than 2%, but have not entered the refractory stage.” The progression from pre-cachexia to cachexia and refractory cachexia is influenced by several factors, such as the type of underlying malignancy, its duration, its stage, its treatment, and their complications (e.g. oral mucositis, nausea, vomiting, diarrhea) (11). The European Society for Clinical Nutrition and Metabolism (ESPEN) expert group emphasized three key steps to update nutritional care for people with cancer: i) screen all patients with cancer for nutritional risk early in the course of their care, regardless of body mass index and weight history; ii) expand nutrition-related assessment practices to include measures of anorexia, body composition, inflammatory biomarkers, resting energy expenditure, and physical function; iii) use multimodal nutritional interventions with individualized plans, including care focused on increasing nutritional intake, lessening inflammation and hypermetabolic stress, and increasing physical activity (1). Thus, patients’ nutritional assessment before, during and after allo-HSCT is extremely important, particularly when infections or GVHD develop and the early recognition of a patient’s malnutrition allows to start effective measure as soon as possible. A prompt treatment for malnutrition is important for achieving resolution of this symptom, whereas a delayed will not be different from palliation (1, 2, 12).

The key point is how to measure patients’ malnutrition, considering that most of the biochemical parameters potentially associated with malnutrition poorly correlate with the nutritional status in the transplant setting (1). To better measure malnutrition, several biomarkers and clinical outcome assessments have been investigated. To date anthropometric biomarkers, such as BMI or brachial circumferences are useful but insufficient to define their clinical role (1, 13).

This is the main reason why the Academy of Nutrition and Dietetics (AND) recommends an early and dynamic evaluation of nutritional status in cancer patients with Patient Generated Subjective Global Assessment (PG-SGA). PG-SGA is a Patient Reported Outcomes (PRO, a clinical outcome assessment according to NIH criteria) tool and it is divided into two sections: the first one is filled in by the patient about subjective sensations on food intake, weight loss perception, nausea, vomiting, dysgeusia, performance status, and daily activities. The second section is filled in by the dietitian (or nurse-specialist) measuring anthropometric and clinical data. The cumulative score defines the threshold for a nutritional intervention. The higher the score, the worse the nutritional status (14).

This tool is considered as the gold standard for nutritional assessment in patients with cancer, and, considering the lack of validated instruments for nutritional assessment in patients subjected to allo-HSCT, it could be reasonably applied to transplanted patients too (15–17).

Another method to identify malnourished transplanted patients is NFPE (Nutrition Focused Physical Exam), also recommended by AND and ASPEN (American Society for Parenteral and Enteral Nutrition) to identify fat and muscle wasting (18) but, in the allo-HSCT setting tested only on a pediatric population (19).

In summary, malnutrition evaluation after allo-HSCT could be influenced by the tool adopted for screening or assessment. Therefore, the integration of biomarkers studies could help in the management of malnutrition in this complex patients setting.

Biomarkers are defined according to the NIH-FDA biomarker working group (20) as “a defined characteristics that are measured as an indicator of normal biological processes, pathogenic processes or responses to an exposure or intervention.” The concept of biomarker is complementary, but distinct, to the concept of Clinical Outcome Assessment (COA), defined as “direct measures of how a person feels, functions or survives.”

Biomarkers are distinguished in subcategories: diagnostic, monitoring, response-pharmacodynamic, predictive, prognostic, safety, susceptibility/risk biomarkers. A recent systematic report (21) identified several biomarkers predictive for malnutrition in older adults.

The aim of this review is to collect the published data on the available diagnostic, monitoring, predictive, or prognostic biomarkers of genomic, proteomic, or metabolomic origin for nutritional assessment before and during allo-HSCT and to better define their role in predicting transplant outcome (1).

The role of identified biomarkers will be discussed according to clinical outcomes, literature data, pathogenesis of gastrointestinal complications, and their management and according to the management of malnutrition after allo-HSCT.

## Methods

A systematic review of original studies has been conducted according to the Preferred Reporting Items for Systematic review and Meta-Analyses Protocols (PRISMA-P) 2009. The search in the database search took place between October and November 2019.

To this purpose, all available studies concerning the use of biomarkers in the field of malnutrition evaluation in allo-HSCT patients were taken into consideration. Further research was performed for biomarkers of metabolic, gastrointestinal, and immunological complications of allo-HSCT. Biomarkers identified by Zhang (20) were included in the literature research and no limit in the type of study design was considered.

Anthropometric biomarkers and COA were excluded from the electronic search.

The databases consulted were Cochrane Central Register of Controlled Trials, MEDLINE, EMBASE, with no limits of time period and only English written literature was selected.

After removing duplicate records, the initial screening of titles and abstracts was conducted independently by two of the authors and excluded those that did not meet the screening review inclusion criteria. Results were reviewed by a senior analyst for authentication and resolution of disagreements between the reviewers. The risk of bias in the studies was assessed using the Cochrane risk-of-bias tool for randomized trials (RoB 2) and Risk of Bias in Non-Randomized Studies-of Interventions (ROBINS-I). Finally, the bibliography articles of all included studies were evaluated. Data were extracted from each relevant publication on study design (study setting, data source, study period), patient characteristics (sample size, mean age, sex).

## Results

**Figure 1:**
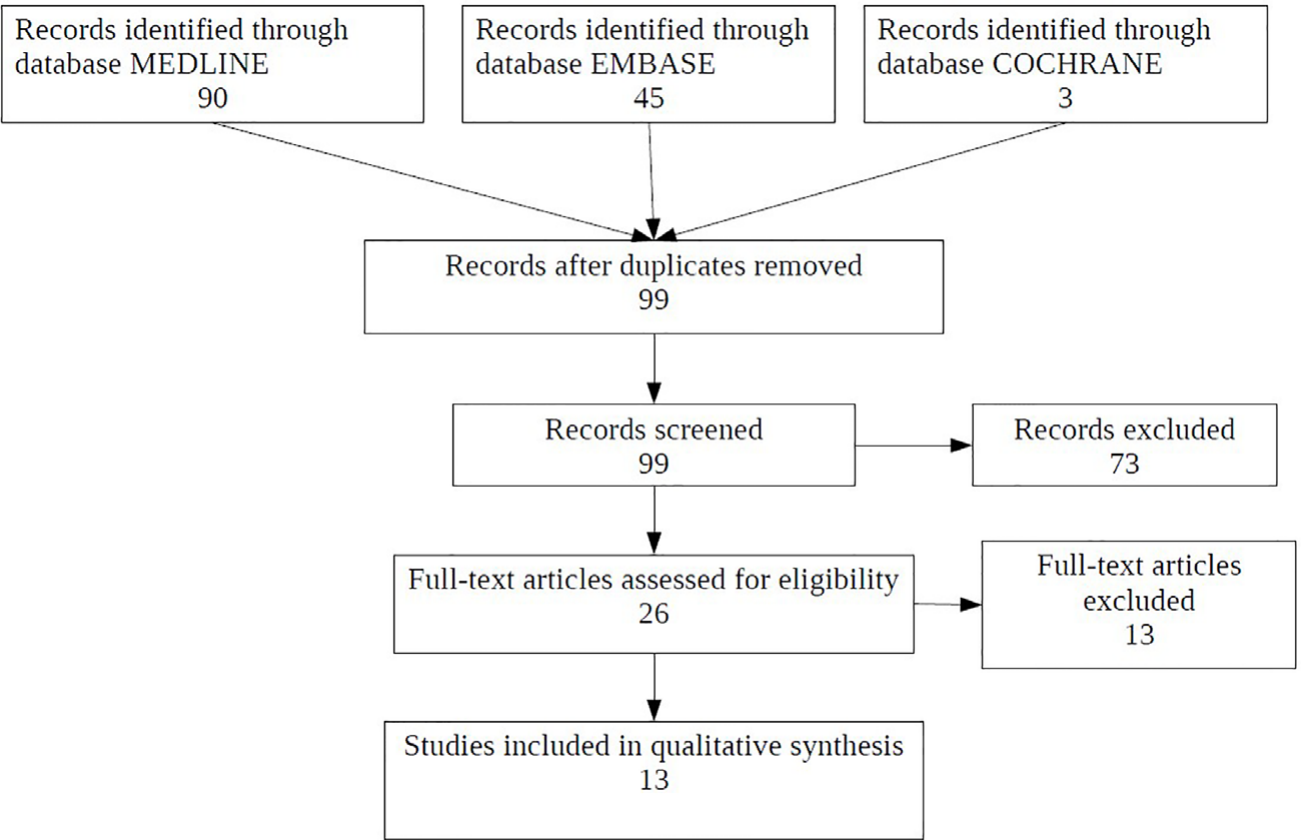
Flow diagram of included studies.

Through the systematic review 13 articles published between 2007 and 2019 were identified and reported in **Figure 1**. All of the studies were observational, with a population range between 14 and 191 and range of age from 0 to 69 years.

The listed studies were divided into two groups. The first group included studies whose primary outcome was the identification of a biomarker for the nutritional assessment of the transplanted patient. The second group considered studies whose primary outcome was to identify biomarkers of nutritional interest for transplant related outcomes. A number of studies were present in both groups (22).

### Biomarkers for Nutritional Status Outcomes

Three studies were selected for these outcomes. The identified biomarkers were transferrin (23, 24), prealbumin (22, 23), albumin (22–24), total urinary nitrogen (22), total proteins (24). Among these studies:

Espinoza et al. (22) included 32 patients who underwent allo-HSCT. Studied biomarkers were prealbumin, prealbumin, total urinary nitrogen, triglycerides. The analysis identified lower albumin and pre-albumin and higher triglycerides as potential useful biomarkers for nutritional related outcomes: despite these findings, BMI was not affected in allo-HSCT setting.

Rzepecki et al. (23) included 24 patients who underwent allo-HSCT. Studied biomarkers were prealbumin, transferrin, and the acute phase proteins. The final analysis also included 30 autologous transplanted patients (total number 54) and identified lower prealbumin and transferrin as useful biomarkers to start nutritional support (Total Parenteral Nutrition in this study).

Krawczyk et al. (24) included 38 patients who underwent allo-HSCT. Studied biomarkers were albumin, total proteins, transferrin, cholesterol, triglycerides, and C-reactive protein. The final analysis, also including 62 autologous transplanted patients (total number 100) identified lower albumin, total proteins, and transferrin as useful biomarkers for malnutrition, worsening of performance status, and mucositis onset.

### Biomarkers for Transplant-Related Outcomes

Ten studies identified biomarkers of nutritional interest for transplant related outcomes (GVHD, gastrointestinal function, mucositis, SOS, overall survival, and transplant related mortality). Identified biomarkers were those of proteomic origin such as citrulline (25–29), albumin (25, 26, 30, 31), prealbumin, and total urinary nitrogen (22) and some of metabolomic origin such as 25-OH-D Vitamin (30), 2-aminobutirric acid, 1-monopalmitin dyacilglycerol, and short chain fatty acids (32).

#### Biomarkers of Proteomic Origin

The retrospective study of Sivgin et al. (31) included 102 patients who underwent allo-HSCT. Studied biomarkers were albumin, fibrinogen, D-Dimer, and serum creatinine. Only Albumin was associated with transplant related outcome: patients with low albumin (<3.2 g/dl) showed a shorter median overall survival (230 days) in comparison to patients with higher albumin levels (570 days, p = 0.007). Infection was an independent factor affecting survival at the multivariate analysis.

In the prospective study of Espinoza 2016 et al. (22) the nutritional outcome section reported the impact of several biomarkers on transplant related outcomes but none of them were associated with overall survival. Indirect outcomes, such as hospital-stay and platelet engraftment were significantly longer in patients with reduced albumin levels 10 days after transplant, and time to platelet engraftment was also associated with increased total urinary nitrogen.

Weischendorff et al. (33) in their prospective study evaluated 23 Plasma Amino Acids (PAA) together with CRP and IL-6 before and after allo-HSCT in 80 patients (age range 1.1–55.4 years). Lower levels of total mean PAA were associated with SOS (Sinusoidal Obstruction Syndrome) and severe acute GVHD. In particular lower levels of glutamic acid, serine, arginine, glycine, lysine, valine, tryptophan, threonine, and proline on day +7 (all p < 0.05) were associated with SOS and serine, glutamine, cysteine, glycine, lysine, and threonine on day +7 (all p < 0.05) were associated with severe acute GVHD.

Serum citrulline levels before allo-HSCT were measured in 191 patients and retrospectively analyzed by Hueso et al. (29) In multivariable analysis low levels of serum citrulline (<26 *µ*Mol/L) before conditioning were associated with severe acute GVHD and transplant related mortality.

Rashidi et al. (28) retrospectively studied potential biomarkers of enteral origin in 95 consecutive allo-HSCT patients to define association with GVHD. Lower pre-transplantation citrulline was associated with severe acute GVHD (p = 0.02). Higher levels of Reg3a at 7 days after transplantation were associated with worse non relapse mortality (p = 0.001).

In the study of Gosselin et al. (27), citrulline levels were studied prospectively in a multi-center cohort of 26 children and correlated with gastrointestinal function after allo-HSCT. Mean citrulline level was 22.7 *µ*Mol/L before conditioning, decrease after transplantation and return to baseline before discharge. Lower levels of citrulline were associated with GVHD (p = 0.0025), reduced oral energy intake (p = 0.018), and severe mucositis (p = 0.003).

In other study, Van der Velden et al. (26) evaluated serum citrulline and albumin levels collected in 48 auto-HSCT and 58 allo-HSCT patients. In this prospective study a graphic analysis of albumin citrulline and CRP was performed and citrulline was suggested as better biomarker for GI complications, specifically mucositis.

Finally, Merlin et al. (25) evaluating plasma citrulline and albumin levels collected and prospectively analyzed in 31 pediatric patients referred to allo-HSCT found that serum citrulline lower than 10 *µ*Mol/L was associated with GI acute GVHD (p = 0.003).

#### Biomarkers of Metabolomic Origin

Contaifer et al. (32) studied a lipidomic and metabolomic profile with LCMS and GCMS before transplantation in 14 patients who underwent allogeneic or autologous transplantation after myeloablative conditioning. The time of sampling was at the end of conditioning before transplantation. Five metabolic biomarkers seem to be predictive for GVHD: 2-aminobutyric acid, 1-monopalmitin, diacylglycerols (DG 38:5, DG 38:6), and fatty acid FA 20:1.

#### Composite Biomarkers

A composite nutritional score including anthropometric data and albumin was retrospectively developed by Kerby et al. (30) to define transplant related complications. The population included 134 pediatric patients (age 0–20.4 years) transplanted from an allogeneic matched or mismatched donor for malignant or non-malignant diseases. The score was proposed in order to increase sensitivity in the identification of patients at risk of malnutrition and was defined retrospectively as any of albumin <2.8 g/dl, weight loss >10% from pre-transplant baseline, BMI <25th percentile or <5th percentile (Score NUT25 and NUT5 respectively). The composite score NUT5 or NUT25 predicted a significant increase (3- to 4-fold) in severe acute GVHD. The score NUT25 predicted also day100 mortality.

## Discussion

In this review data from 13 trials, published between 2007 and 2019, were collected. Overall, 829 patients were included: 522 and 307 were retrospective and prospective trials, respectively; only 14 in prospective validation studies.

As a result of our investigation, no reliable biomarker has been identified as “gold standard” for the assessment of nutritional status in allo-transplanted patients.

Several identified biomarkers in this analysis, such as albumin and citrulline, are not directly associated with malnutrition, but are crucial in the transplanted patients’ metabolism and were studied for transplant related outcomes that are strongly linked to gastrointestinal failure such as mucositis or GVHD.

**Figure 2:**
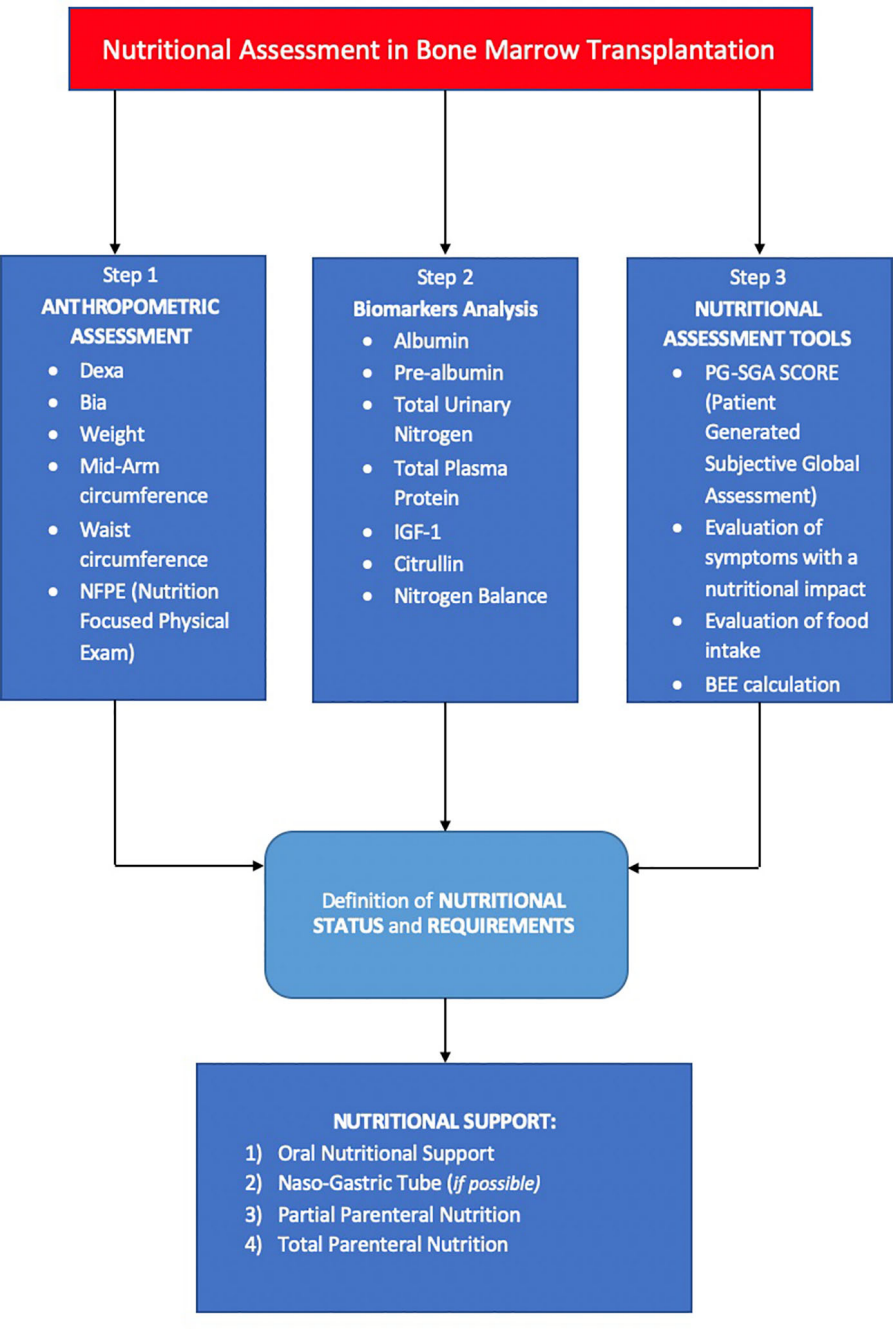
Shows the three steps for nutritional assessment in bone marrow transplantation. First step includes an objective evaluation of anthropometric aspects. The second step involves an investigation on nutritional biomarkers. The third step includes tools that allow to standardize nutritional assessment. Thanks to these three steps, it is possible to make a nutritional diagnosis and start an earlier and personalized nutritional support.

In a multidimensional approach, nutrition and immune-system interactions should be studied with an -omics integration including genomic (on patients, donors, and microbiota), proteomic (from immune-system and enteral origin), metabolomic (at the several metabolic processes of enteral, immune, and endocrine-metabolic functions), and finally patient-derived outcomes (34) (**Figure 2**).

Of interest, no genomic studies were reported in this review: to date, more research is needed to define the complex interaction between the genetic determinants of protein metabolism, malnutrition, and immune system function, particularly in the transplant setting. Nevertheless, genomic studies should also include microbiota composition, although costs of such research are higher than proteomic studies.

**Figure 3:**
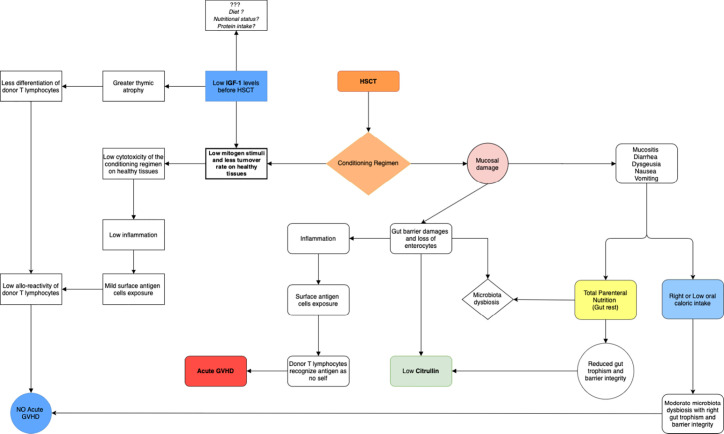
Shows the possible role of IGF-1 in the development of acute GVHD and the possible role of citrulline in demonstrating the presence of mucosal damage. The conditioning regimen causes a mucosal and gut barrier damage. Gut toxicity has a huge impact on nutritional status. Mucosal and gut barrier damage could be evaluated by plasma citrulline levels: a biomarker of gut barrier damage. Insulin-like growth factor-1 (IGF-1) is the major mediator of growth hormone (GH), as well as a mediator of GH-independent anabolic responses in many cells and tissues. Low levels of IGF-1 in pre-transplant period, seems to be associated with a lower risk of acute GVHD: a low mitogen stimuli on healthy tissues, mediated by low IGF-1 levels, could reduce damages and tissues inflammation mediated to chemotherapy, but this hypothesis should be confirmed by larger studies.

Focusing on the selected studies included in the present review, several protein derived biomarkers were identified: pre-albumin, albumin, total urinary nitrogen, and total plasma proteins appear as potentially useful biomarkers for the indirect assessment of nutritional status in patients addressed to allo-HSCT. Albumin was correlated with overall survival (31), the duration of hospital stay, with platelets engraftment (22) and with GVHD (30). Transferrin seems to be a useful biomarker too, but it is not easily reliable in allo-HSCT, because patients are always hyper-transfused and often present an iron overload. Other parameters proposed as biomarkers in allo-transplanted patients include citrulline, which is related to gut integrity and permeability (27) and in some studies (more than 400 patients) seems to be associated to aGVHD (28), in particular intestinal aGVHD (25) and mucositis (26) (**Figure 3**). Citrulline is not a specific malnutrition biomarker, although its role in evaluating intestinal failure is crucial (35). In a proteomic view, several proteins and/or amino acids could be tested together to better define these complex interactions, but to date no proteomic studies are available in this setting.

A single but important study by Contafier (32) brought interesting results in metabolomic and lipidomic patterns of 14 allo-transplanted patients. Five metabolic biomarkers seem to be predictive for GVHD and this approach should be validated on larger numbers.

A new parameter, that has not been extensively studied in this setting as malnutrition biomarkers yet, is IGF-1. IGF-1 is produced from in the liver, following GH stimulation. Although pre-transplant low IGF-1 was reported as associated with fluid retention and Sinusoidal Obstruction Syndrome (SOS) (36), in a cohort of 330 patients it seems to be significantly associated with GVHD (37). One explanation could be that IGF-1 is a factor that stimulate cell cycle in an anabolic direction and high level of IGF-1 before allo-HSCT may increase the risk of GVHD. In detail the hyperproliferation of healthy tissue induced by IGF-1 may expose the cells to a strong cytotoxic effect of chemo-radiotherapy administered during conditioning and of inflammation that is subsequently present. Damaged healthy tissues may, thus, increase the exposition of self-antigens that may drive the donor immune response (**Figure 3**) in this view IGF-1 could be a potentially useful biomarker of the complex interaction between tissue damaging, enteral, metabolic, and immune-system function.

## Conclusions

An extreme variability of the nutritional approach to the transplant patient is reported among European allo-HSCT centers (38) and there is the need for a patient centered approach and more research in this field. The nutritional status in patients subjected to allo-HSCT should be assessed by combining anthropometric data (e.g. DEXA, BIA, direct or indirect calorimetry), biochemical markers, and questionnaires collecting patients’ reported outcome (PRO), such as the Patient-Oriented Subjective Global Assessment (PG-SGA score), or dietary intake.

The PG-SGA score, a “clinical outcome assessment” according to NIH criteria (20), is recognized as the “gold standard” for the evaluation of the nutritional status in oncology.

To date, no studies defines malnutrition according to GLIM (Global Leadership Initiative on Malnutrition) criteria in the setting of allo-HSCT (39) and there are no other validated and reliable instruments for an easy, dynamic, and standardized assessment of malnutrition in allo-HSCT, a tool like PG-SGA appears very attractive for this topic if regularly used by dieticians, nurses, and hematologists. Nevertheless, data derived from PG-SGA should be integrated with metabolic and anthropometric assessment of nutritional status (e.g. DEXA or BIA), before and after allo-HSCT. In fact, DEXA and BIA are particularly useful in the measure of body composition in terms of fat and muscular mass and basal metabolism, any time during allo-HSCT, to define the caloric requirement of each patient together with calorimetry. Some studies focused on the modification of energetic metabolism during allo-HSCT and suggested that the loss of muscular mass associated with allo-HSCT produces a reduction in basal metabolism, that induces an over-support with intravenous nutrition. Thus, an extensive and dynamic assessment of the basal metabolism and calorie requirement is crucial to personalize the nutritional support, which varies depending on the patient. During the aplastic phase, BIA seems to be the most reliable instrument for the assessment of nutritional status, as the body weight is highly variable and influenced by different factors, such as intravenous hydration, every day. More research is needed to better define the ideal combination of malnutrition biomarkers (e.g. citrulline) in relation to immune system function together with anthropometric assessment and PRO.

## Author Contributions

EM, MGG, FA, and MM designed the review. EM and MGG revised the literature and collected the data. EM, MGG, FA, and MM wrote the article. MA, DR, SB, AT and NP revised the manuscript and contributed to the discussion. All authors contributed to the article and approved the submitted version.

## Conflict of Interest

The authors declare that the research was conducted in the absence of any commercial or financial relationships that could be construed as a potential conflict of interest.
